# Outcomes in deprescribing implementation trials and compliance with expert recommendations: a systematic review

**DOI:** 10.1186/s12877-023-04155-y

**Published:** 2023-07-12

**Authors:** Pierre Nizet, Adrien Evin, Emma Brociero, Caroline Victorri Vigneau, Jean-François Huon

**Affiliations:** 1 Nantes Université, CHU Nantes, 44000 Pharmacie, France; 2 U1246 SPHERE “methodS in Patient-Centered Outcomes and HEalth ResEarch”, Université de Nantes, Université de Tours, INSERM, Nantes, France; 3 Nantes Université, CHU Nantes, Service de Soins Palliatifs Et de Support, 44000 Nantes, France; 4 Nantes Université, CHU Nantes, Service de Pharmacologie Clinique, 44000 Nantes, France

**Keywords:** Deprescribing, Outcome assessment, Polypharmacy, Review, Potentially inappropriate medications

## Abstract

**Background:**

Deprescribing, defined as discontinuing or reducing the dose of medications that are no longer needed or for which the risks outweigh the benefits is a way to reduce polypharmacy. In 2022, the US Deprescribing Research Network (USDeN) published recommendations concerning the measurement of outcomes for deprescribing intervention studies. The objectives of this systematic review were to identify the outcome categories used in deprescribing intervention trials and to relate them to the previously published recommendations.

**Methods:**

We searched MEDLINE, Embase, PsychInfo, and the Cochrane library from January 2012 through January 2022. Studies were included if they were randomized controlled trials evaluating a deprescribing intervention. After data extraction, outcomes were categorized by type: medication outcomes, clinical outcomes, system outcomes, implementation outcomes, and other outcomes based on the previously published recommendations.

**Results:**

Thirty-six studies were included. The majority of studies focused on older adults in nursing homes and targeted inappropriate medications or polypharmacy. In 20 studies, the intervention was a medication review; in seven studies, the intervention was educational or informative; and three studies based their intervention on motivational interviewing or patient empowerment. Thirty-one studies presented a medication outcome (primary outcome in 26 studies), 25 a clinical outcome, 18 a system outcome, and seven an implementation outcome. Only three studies presented all four types of outcomes, and 10 studies presented three types of outcomes.

**Conclusions:**

This review provides an update on the implementation of gold standard deprescribing studies in clinical practice. Implementation outcomes need to be developed and specified to facilitate the implementation of these practices on a larger scale and clinical outcome need to be prioritized. Finally, this review provides new elements for future real-life deprescribing studies.

**Supplementary Information:**

The online version contains supplementary material available at 10.1186/s12877-023-04155-y.

## Introduction

### Deprescribing: a topical issue

Deprescribing has become a priority today because polypharmacy can increase the risk of drug-related problems [1–8]. Deprescribing can be defined as a patient-centered process conducted under the supervision of a healthcare professional for discontinuing or reducing the dose of medications that are no longer needed, for which the risks outweigh the benefits, or that are incompatible with the goals of care [9–11]. In deprescribing implementation trials, an intervention (educational intervention, medication review, motivational interviewing, etc.) is applied to one or more participants to increase deprescribing. The main objective of these studies is generally to assess the success or not of the intervention. Clinical outcomes are usually secondary, depending on the success of the intervention as well as unintended benefits that were not a direct result of deprescribing [11]. These trials are to be distinguished from medication cessation trials in which all participants in the intervention group stop taking the deprescribed drugs. These trials provide direct information on the clinical benefits and the harm of deprescribing in the target population.

### Heterogeneous methodologies, new recommendations

Many systematic reviews have focused on assessing the impact of deprescribing in a targeted population, such as older adults [12–14], on a specific type of medication, such as drugs that increase the risk of falls (FRIDs) or benzodiazepines (BZD) and benzodiazepine-related drugs [15–17], or on a specific outcome, such as compliance [18]. The conclusions of these systematic reviews are consistently limited because of a large heterogeneity when it comes to comparing deprescribing interventions and their methodologies.

In 2022, following the recommendations of Aubert et al. published two years earlier [19], the US Deprescribing Research Network (USDeN) published guidelines concerning the measurement of outcomes for deprescribing intervention studies [20]. These recommendations take the form of a conceptual framework that includes different categories of outcomes to be followed in deprescribing implementation studies especially when it comes to performing randomized clinical trials, considered the gold standard in research: medication outcomes that directly reflect the deprescribing intervention by quantifying changes in the total number or dose of drugs, clinical outcomes that reflect the downstream effects of drug reduction on patients, system outcomes that reflect population-level effects (hospitalization, quality of care …) and finally implementation outcomes such as effectiveness and setting that are essential for large-scale implementation of interventions. The objectives of this review were *(i)* to identify the outcome categories used in deprescribing implementation trials over the past 10 years and *(ii)* to relate them to the previously published recommendations.

## Methods

### Study design

A review of the literature was conducted to select randomized controlled trials evaluating a deprescribing implementation intervention. The outcomes used in each study were identified and categorized according to the classification proposed in the recommendations of the US Deprescribing Research Network (USDeN) concerning the measurement of outcomes for deprescribing intervention studies [20]. These recommendations are proposed in the form of a conceptual framework that includes different categories of outcomes: medication outcomes that directly reflect the deprescribing intervention by quantifying changes in the total number or dose of drugs, clinical outcomes that reflect the downstream effects of drug reduction on patients (function, quality of life, adverse drug withdrawal events, etc.), system outcomes that reflect population-level effects (hospitalization, quality of care, cost of care, etc.), and finally implementation outcomes such as the effectiveness and setting that are essential for large-scale implementation of interventions (reach, effectiveness, adoption, etc.).

### Systematic review

- **Protocol and registration**

The protocol of this review was registered on PROSPERO (CRD42022360796) and was conducted in compliance with the Preferred Reporting Items for Systematic Review and Meta-Analysis Extension for Systematic Reviews (PRISMA) Additional file 2: Appendix 2 [21].

- **Search strategy**

The search strategy was developed with a senior librarian. We searched MEDLINE from January 2012 to January 2022 using Medical Subject Headings (MeSH) and keywords for deprescribing. Keywords were selected when they were synonymous with deprescribing, stopping or reducing medication. We used terms ceas*, cessation, decreas*, deprescrib*, de-prescrib*, de-prescrip*, discontinu*, eliminate*, reduc*, stop*, taper*, substitut*, withdraw*, optimiz*, remov*, interrupt*, step-down*, restriction, deintensification, diminish* and drop* in the research equation. Embase, PsychInfo, and the Cochrane Central Register of Controlled Trials electronic databases were searched using a strategy based on the MEDLINE strategy. This strategy was adapted for searching in other databases. The MEDLINE search strategy is available in Additional file 1: Appendix S1.

- **Study eligibility criteria**

Three investigators independently evaluated each title first, then each abstract among the selected articles, which moved to full-text review if two investigators considered the citation eligible. Disputes were resolved by discussion with input from a fourth investigator if needed.

Studies were included if they were randomized controlled trials evaluating a deprescribing implementation intervention. The comparator was defined as usual care.

Excluded studies were protocols, qualitative studies, medico-economic studies, conference abstracts, academic theses, commentaries, and opinion articles, or that were not published in English and for which the full text was not available. In addition, medication cessation trials were excluded, *i.e*., when one or more drug was discontinued in all patients in the intervention arm.

### Outcomes categorization according to the new recommendations

Two investigators independently extracted data regarding the study characteristics. Disagreements were resolved through consensus and a thorough review of the article. The extracted data were as follows: author, publication year, country, target population (study location and minimum age), target medication, intervention type, duration of the follow-up, and outcomes.

After data extraction, the outcomes were categorized by type based on the previously published recommendations [20]: medication outcomes, clinical outcomes, system outcomes, implementation outcomes, and other outcomes. The primary outcome was identified in each study and the studies were classified alphabetically by the author’s name.

## Results

### Study selection

**Fig. 1:**
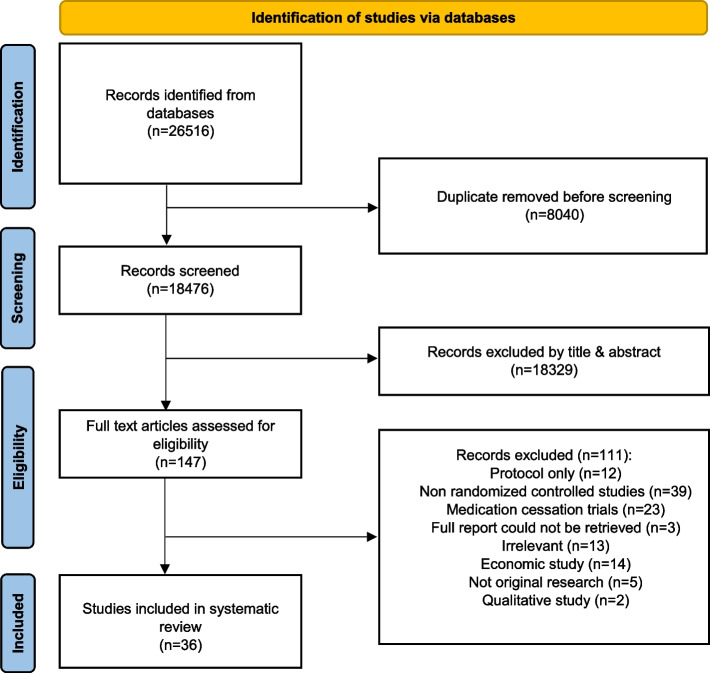
PRISMA Flow Diagram of identification studies

A total of 26 516 records were retrieved from the databases. After the removal of duplicates, 18 476 titles, and abstracts were screened for eligibility. Full-text articles were sought and screened, yielding 147 eligible articles. Ultimately, 36 studies were included [22–57] (Fig. 1).

### Characteristics of the included studies

#### Participants and settings

The 36 included studies took place in 16 different countries: 19 (52.8%) in Europe [22, 25, 28–33, 35, 36, 38, 41, 46, 48, 52–54, 56, 57], 11 (30.6%) in North America [23, 24, 26, 27, 34, 39, 43, 44, 49–51], four (11.1%) in Asia [40, 42, 47, 55], and two (5.6%) in Australia [37, 45]. The general characteristics of the selected studies are presented in Table 1.

**Table 1:** General characteristics of included studies

Author	Publication year	Country	Target population		Target medication	Intervention type	Duration maximum of the follow-up period (months)	
			Study location	Minimum age (years)				
Aharaz A. [22]	2021	Denmark	Subacute medical outpatient	18	PIMs	Medication drug review (pharmacist)		12
Ashworth N. [23]	2021	Canada	Primary care	65	BZD	Patient information		12
Balsom C. [24]	2020	Canada	Residents in nursing home	65	PIMs	Medication drug review (pharmacist)		6
Boyé NDA. [25]	2017	Netherlands	Hospital, emergency department	65	FRID	Medication drug review (research team)		12
Campbell NL. [26]	2019	USA	Hospital, intensive care unit	NA	BZD and anticholinergic	Computerized decision support intervention + pharmacist		1
Campbell NL. [27]	2021	USA	Primary care	65	Anticholinergic	Medication drug review (multidisciplinary)		12
Cateau D. [28]	2021	Switzerland	Residents in nursing home	65	PIMs	Medication drug review (pharmacist)		4
Cateau D. [29]	2021	Switzerland	Residents in nursing home	NA	PIMs	Medication drug review (pharmacist)		12
Clyne B. [30]	2016	Ireland	Primary care	70	PIMs	Medication drug review (multidisciplinary)		12
Cool C. [31]	2018	France	Residents in nursing home	NA	PIMs	Geriatric intervention		18
Curtin D. [32]	2020	Ireland	Residents in nursing home	75	PIMs	Medication withdrawal plan communicated to the patient's physician		3
Dalleur O. [33]	2014	Belgium	Hospital, medical ward	75	PIMs	Medication drug review (geriatrician)		12
Edey R. [34]	2019	Canada	Hospital, clinical teaching unit	19	PIMs	Medication drug review (pharmacist)		1
Eveleigh R. [35]	2017	Netherlands	Primary care	NA	Antidepressant	GP deprescribing (after assessing the appropriateness of the deprescribing attempt)		12
Gedde MH. [36]	2021	Norway	Residents in nursing home	65	Psychotropic drugs	Medication drug review (multidisciplinary)		4
Gnjidic D. [37]	2019	Australia	Hospital, medical ward	65	BZD	Patient information		1
Gulla C. [38]	2018	Norway	Residents in nursing home	65	Antihypertensive drugs	Medication drug review (multidisciplinary)		9
Hah JM. [39]	2020	USA	Hospital, orthopedic surgery unit	18	Opioid	Motivational interviewing with patient		12
Ee C.[40]	2018	Singapore	Rehabilitation hospital	NA	Symptomatic medication	Medication drug review (pharmacist)		1.5
Kersten H. [41]	2013	Norway	Residents in nursing home	NA	Anticholinergic	Medication drug review (multidisciplinary)		4
Kua CH. [42]	2021	Singapore	Residents in nursing home	65	PIMs	Medication drug review (pharmacist)		12
Kuntz JL. [43]	2019	USA	Primary care	64	BZD-related drugs	GP and patient information		6
Navy HJ. [44]	2018	USA	Primary care	65	BZD	Patient information		6
Potter K. [45]	2016	Australia	Residents in nursing home	65	PIMs	Medication drug review (pharmacist)		12
Rieckert A. [46]	2020	Austria, Italy, United Kingdom	Primary care	75	PIMs	Computerized decision support tool providing a comprehensive drug review		24
Sathienluckana T. [47]	2018	Thailand	Outpatient department of psychiatry	18–50	Anticholinergic	Pharmacist deprescribing		3
Smeets CHW. [48]	2020	Netherlands	Residents in nursing home	NA	Psychotropic drugs	Medication drug review (multidisciplinary)		18
Sullivan MD. [49]	2017	USA	Primary care	NA	Opioids	Patient information + motivational interviewing with patient		8.5
Tannenbaum C. [50]	2014	Canada	Primary care	65	BZD	Patient empowerment intervention		6
Tseng ES. [51]	2021	USA	Hospital, trauma unit	NA	Opioid	Patient information		1
Van der Meer HG. [52]	2018	Netherlands	Primary care	65	Anticholinergic and sedative drugs	Medication drug review (pharmacist)		3
Vicens C. [53]	2014	Spain	Primary care	NA	BZD	GP information		12
Vicens C. [54]	2016	Spain	Primary care	NA	BZD	GP information		36
Wong APY. [55]	2021	Singapore	Rehabilitation hospital	65	PIMs	Medication drug review (multidisciplinary)		At the discharge
Wouters H. [56]	2017	Netherlands	Residents in nursing home	NA	PIMs	Medication drug review (multidisciplinary)		4
Zechmann S. [57]	2020	Switzerland	Primary care	60	PIMs	Medication drug review (GP)		12

Abbreviations: *BZD* Benzodiazepines, *FRID* Fall-risk increasing drugs, *GP* General practitioner, *NA* Not applicable, *PIMs* Potentially inappropriate medications, *USA* United States of America

### The majority of studies were on older adults in institutions, and the duration of the follow-up period was heterogenous

The majority of studies (26; 72.2%) specifically targeted older adults, either by age criteria [23–25, 27, 28, 30, 32, 33, 36–38, 42–46, 50, 52, 55, 57] or by the use of tools for identifying inappropriate drugs in older adults [22, 24, 25, 28–34, 42, 45, 46, 55–57]. In 18 studies (50%), the patients were over 65 years of age [23–25, 27, 28, 30, 32, 33, 36–38, 42, 44–46, 50, 52, 55]. Regarding the setting, 13 studies (36.1%) took place in primary care [23, 27, 30, 35, 43, 44, 46, 49, 50, 52–54, 57], 12 (33.3%) in nursing homes [24, 28, 29, 31, 32, 36, 38, 41, 42, 45, 48, 56], seven (19.4%) in hospitals (emergency department, surgery unit, intensive care unit, trauma unit) [25, 26, 33, 34, 37, 39, 51], two (5.6%) in a rehabilitation hospital [40, 55], one (2.8%) in an outpatient department of psychiatry [47], and one (2.8%) in subacute medical outpatient [22]. The follow-up period ranged from one to 36 months, with 13 studies (36.1%) with a duration of follow-up of 12 months [22, 23, 25, 27, 29, 30, 33, 35, 39, 42, 45, 53, 57] and four studies (11.1%) with a follow-up period longer than 12 months [31, 46, 48, 54].

### Targeting inappropriate medication and polypharmacy in older adults

In most cases, the studies were conducted to address the prevalence of inappropriate medications or to reduce polypharmacy in older adults. Regarding the drugs targeted by the deprescribing intervention, the results were heterogeneous. Indeed, 15 studies (41.7%) targeted potentially inappropriate medications (PIMs) [22, 24, 28–34, 42, 45, 46, 55–57] in older adults using different lists or criteria (STOPP criteria [58, 59], Beer’s list [60], Laroche list [61], or a country-specific list). Other studies targeted specific drugs such as benzodiazepines and BZD-related drugs (*n* = 7; 19.4%) [23, 37, 43, 44, 50, 53, 54], anticholinergic drugs (*n* = 3; 8.3%) [27, 41, 47], psychotropic drugs (*n* = 2; 5.6%) [36, 48], or opioids (*n* = 3; 8.3%) [39, 49, 51].

### Using medication reviews in a multidisciplinary context

In 20 studies, the intervention was a medication drug review, carried out most of the time by a pharmacist (*n* = 9; 25%) [22, 24, 28, 29, 34, 40, 42, 45, 52] or a multidisciplinary team (*n* = 8; 22.2%) [27, 30, 36, 38, 41, 48, 55, 56]. In seven studies (19.4%), the intervention was educational or informative, provided to either patients (*n* = 5; 13.9%) [23, 37, 44, 49, 51] or general practitioners (*n* = 2; 5.6%) [53, 54], or both (*n* = 1; 2.8%) [43]. In two other studies (5.6%), the deprescribing intervention was carried out using computerized decision support for physicians [26, 46]. Three studies based their intervention on motivational interviewing or patient empowerment [39, 49, 50].

### Outcomes of the included studies

Among the 36 included studies, 31 (86.1%) presented a medication outcome [22–24, 27–36, 38–40, 42–46, 48–57], 25 (69.4%) a clinical outcome [24–26, 28, 29, 32, 33, 35, 36, 38–42, 45–49, 52–57], 18 (50%) a system outcome [23, 25, 26, 28–30, 32, 34, 38, 40, 42, 43, 45, 46, 51, 55–57], and seven (19.4%) an implementation outcome [22, 34, 37, 40, 49, 55, 57]. The outcome type of the included studies is presented in Table 2, and their characteristics are presented in Tables 3, 4, and 5.

**Table 2:** Outcome type in studies included

Author	Publication year	Medication outcomes	Clinical outcomes	System outcomes	Implementation outcomes	Other outcomes
Aharaz A. [22]	2021	X			X	X
Ashworth N. [23]	2021	X		X		
Balsom C. [24]	2020	X	X			
Boyé NDA. [25]	2017		X	X		
Campbell NL. [26]	2019		X	X		X
Campbell NL. [27]	2021	X				
Cateau D. [28]	2021	X	X	X		X
Cateau D. [29]	2021	X	X	X		X
Clyne B. [30]	2016	X		X		
Cool C. [31]	2018	X				
Curtin D. [32]	2020	X	X	X		
Dalleur O. [33]	2014	X	X			
Edey R. [34]	2019	X		X	X	X
Eveleigh R. [35]	2017	X	X			
Gedde MH. [36]	2021	X	X			
Gnjidic D. [37]	2019				X	X
Gulla C. [38]	2018	X	X	X		
Hah JM. [39]	2020	X	X			
Ee C.[40]	2018	X	X	X	X	
Kersten H. [41]	2013		X			X
Kua CH. [42]	2021	X	X	X		X
Kuntz JL. [43]	2019	X		X		
Navy HJ. [44]	2018	X				X
Potter K. [45]	2016	X	X	X		
Rieckert A. [46]	2020	X	X	X		
Sathienluckana T. [47]	2018		X			X
Smeets CHW. [48]	2020	X	X			
Sullivan MD. [49]	2017	X	X		X	X
Tannenbaum C. [50]	2014	X				
Tseng ES. [51]	2021	X		X		
Van der Meer HG. [52]	2018	X	X			
Vicens C. [53]	2014	X	X			
Vicens C. [54]	2016	X	X			
Wong APY. [55]	2021	X	X	X	X	
Wouters H. [56]	2017	X	X	X		
Zechmann S. [57]	2020	X	X	X	X	X

**Table 3:** Medication outcomes of included studies

Authors	Medication outcomes
Aharaz A. [22]	**Differences in deprescribing rates for patients in the intervention versus control group with > 1 medication deprescribed**
	Change in total number of medications
	Percentage of deprescribed medications and sustained
Ashworth N. [23]	**Mean number of older patients prescribed high dose of BZD**
	**Mean defined daily dose of BZD**
Balsom C. [24]	**Change in the number of prescribed regular and as-needed medications**
Campbell NL. [27]	**Proportion of anticholinergic orders prescribed as discontinuation orders in the preintervention and postintervention periods**
	Population prevalence of anticholinergic use
Cateau D. [28]	**Number of PIMs used**
	Number of chronic drugs
	Number of inappropriate defined daily dose
	Number of chronic defined daily dose
Cateau D. [29]	**Proportion of galenic units considered potentially inappropriate at the follow up**
	**Number of PIMs per day (defined daily dose)**
	**Number of PIMs per resident (defined daily dose/resident)**
	Number of defined daily dose/resident to avoid and to reevaluate
Clyne B. [30]	**Proportion of patients with PIP and mean of PIP**
Cool C. [31]	**Potentially inappropriate drug prescribing**
Curtin D. [32]	**Mean change in the number of long term prescribed medicines**
	Changes in prescription of neuroleptic, antipsychotic medications
Dalleur O. [33]	**Proportion of PIMs discontinued**
Edey R. [34]	**Number of discontinued home medications at hospital discharge**
	Proportion of medication remaining deprescribing at 30 days after discharge
Eveleigh R. [35]	**Proportion of patients discontinuation ATD**
Gedde MH. [36]	**Mean change in numbers of prescribed psychotropic drugs**
Gulla C. [38]	Number of antihypertensive drugs
Hah JM. [39]	**Time to baseline opioid use**
	Time to complete opioid cessation
Ee C.[40]	Reduction in the total number of medications
	Needs for deprescribed medications to be restarted or initiation of new symptomatic control medications after deprescribing
Kua CH. [42]	Pill burden
Kuntz JL. [43]	**Discontinuation of BZD-related drugs**
	Number of Z-drug dispensing
Navy HJ. [44]	**Composite criteria:** **- Rate of patients who had no alprazolam dispensing** **- Rate of patients who had an alprazolam dose reduction** **- Rate of patients who interchanged to an alternate medication**
Potter K. [45]	**Mean change in drugs number**
	Mean change in drugs number
Rieckert A. [46]	Number of drugs prescribed
Smeets CHW. [48]	Prescription of psychotropic drugs
Sullivan MD. [49]	**Mean daily opioid dose in the past week at 22 weeks after randomization**
	Opioid dose 34 weeks after randomization
	Percent reduction from baseline in opioid dose
Tannenbaum C. [50]	**Complete cessation of BZD**
	Dose reduction of BZD
Tseng ES. [51]	**Opioid use on the day prior to discharge**
	Percentage of patients who were discharged with prescriptions for opioid or ancillary medications
	Morphine equivalent doses prescribed at discharge
	Late prescription and morphine equivalent doses at 30 days
Van der Meer HG. [52]	**Difference in proportion of patients having a decrease of DBI > 0,5**
Vicens C. [53]	**Discontinuation BZD**
Vicens C. [54]	**BZD cessation**
Wong APY. [55]	**Percentage reduction of total daily dose of PIMs**
	Percentage reduction of total number of medicine of PIMS
	Medicine reinitialization or substitution
Wouters H. [56]	**Proportion of resident who successfully discontinued use of at least 1 inappropriate medication**
	Number of residents for whom at least 1 underprescribing medication was initiated at least 1 dose was adjusted, and at least 1 potentially hazardous drug was replaced by a safer alternative
	Cumulative exposure to anticholinergic and sedative drugs
Zechmann S. [57]	**Mean difference in the number of drugs per person**
	Number of drug change recommendations and kind of change
	Number of DPP taken without the GP knowledge at pre intervention

Legend: primary outcomes are in bold.

Abbreviations: *ATD* Antidepressants, *BZD* Benzodiazepines, *DBI* Drug burden index, *DPP* Drugs per person, *GP* General practitioner, *PIMs* Potentially inappropriate medications, *PIP* Potentially inappropriate prescribing

**Table 4:** Clinical outcomes of included studies

Authors	Clinical outcomes
Balsom C. [24]	Cognitive performance*, depression*, pain*, social engagement, health status, and activities of daily living
	Survival
Boyé NDA. [25]	Time since the first fall*
	Time since the second fall*
Campbell NL. [26]	Delirium severity*
	Mortality rate
	Falls number*
	Pressure ulcer*
Cateau D. [28]	Number of complaints
	QoL
	Falls number*
	Mortality rate
Cateau D. [29]	Falls number per resident*
	Falls number per year*
	Mortality rate
Curtin D. [32]	Falls number*
	Nonvertebral fracture number*
	QoL
	Death number
Dalleur O. [33]	Clinical relevance
Eveleigh R. [35]	General distress and depressive symptoms*
	Somatic comorbidity*
Gedde MH. [36]	Behavioral and psychological symptom of dementia*
	Activities of daily living
Gulla C. [38]	Blood pressure*
	Death number
Hah JM. [39]	Adverse drug reactions*
	Time to surgical recovery
	Time to pain cessation*
Ee C.[40]	Constipation*
	Other symptom recurrence
	Adverse drug withdrawal events
Kersten H. [41]	Cognitive function*
	Mouth dryness*
Kua CH. [42]	Fall rate*
	Fall risk*
	Functional status
	Mortality rate
	Cognitive status*
Potter K. [45]	Falls number*
	Nonvertebral fracture number*
	Cognitive function*
	Physical function*
	Bowel function*
	QoL
	General health
	Sleep quality*
	Survival
Rieckert A. [46]	**Composite outcome: death + system outcome**
	Mortality cause
	Falls number*
	Recorded fractures number*
	Adverse drug reactions
	QoL
Sathienluckana T. [47]	Cognitive function*
	Psychiatric symptoms*
	Proportions of patients who had a clinical response of psychopathological symptoms*
Smeets CHW. [48]	Neuropsychiatric symptoms*
Sullivan MD. [49]	Pain severity*
	QoL
	Anxiety*
	Insomnia*
	Confidence in ability to do tasks and activities despite pain
	Somatic symptom severity*
	Opioid craving*
	Patient global impression of change
Van der Meer HG. [52]	Anticholinergic side effect*
	Sedative side effect*
	Fall risk*
	Cognitive function*
	Activities of daily living
	QoL
Vicens C. [53]	Anxiety*
	Depression*
	Sleep satisfaction*
	Withdrawal symptoms*
	Alcohol consumption
Vicens C. [54]	Anxiety*
	Depression*
	Sleep satisfaction*
Wong APY. [55]	Recurring or worsering symptoms*
	Death number
Wouters H. [56]	Falls number*
	Cognitive function*
	Neuropsychiatric symptoms*
	QoL
Zechmann S. [57]	Symptom scores*
	Unexpected clinical events
	QoL
	Death number

Legend: primary outcomes are in bold. * means that the clinical outcome measured is directly related to the deprescribed drug

Abbreviations: *QoL* Quality of life

**Table 5:** System, implementation and others outcomes of included studies

Authors	System outcomes	Implementation outcomes	Others outcomes
Aharaz A. [22]		Percentage of eligible patients that agreed to participate in the study	Percentage of patients who completed the study
Ashworth N. [23]	Crude direct costs of each intervention		
Boyé NDA. [25]	Time since the first GP consultation because of fall or emergency department		
Campbell NL. [26]	Length of stay		Pulling out intravenous lines or urinary catheters
			Reintubation
			Use of physical restraints
Cateau D. [28]	Hospitalizations number		Days with physical restraints
	Number of hospital days		
Cateau D. [29]	Number of hospital days		Rate of use of physical restraints
Clyne B. [30]	Health service utilization		
	Number of GP visits		
	Number of hospital days		
Curtin D. [32]	Unscheduled medical reviews		
	Emergency transfers		
	Unplanned hospital admission		
	Change in 28 day cost of participants' prescription medication		
Edey R. [34]	Readmission	Physician impression of deprescribing rounds	Patient perception of deprescribed medications
	Emergency department visit		
Gnjidic D. [37]		Participants attitudes and beliefs towards deprescribing	**Proportion of patients who initiated a discussion with a healthcare professional regarding the withdrawal of their BZD**
Gulla C. [38]	Hospitalization number		
Ee C.[40]	**Cost saving of systematic deprescribing**	Time required to complete the deprescribing process and the limitations and challenges encountered	
Kersten H. [41]			Serum anticholinergic activity
Kua CH. [42]	Cost related measures		Drug related problems
	Hospitalization		Deprescribing acceptance rate
			Number of deprescribing interventions
Kuntz JL. [43]	Hospitalizations number		
	Urgent care and emergency department visits		
Navy HJ. [44]			Rate of intervention patients who called the study CP within 14 days of the study letter being mail
Potter K. [45]	Hospitalizations number		
Rieckert A. [46]	**Composite outcome: Unplanned hospital admission + clinical outcome**		
	Unplanned hospital admission number		
Sathienluckana T. [47]			Frequencies of identified drug related problems
Sullivan MD. [49]		Perceived Helpfulness	Patients difficulties with opioid therapy
Tseng ES. [51]	Patients contacted trauma providers		
	Patients contacted consultants		
	Patients contacted emergency medicine teams		
	Patients contacted other physicians for further pain-related prescriptions		
Wong APY. [55]	Re-hospitalization	Feasibility of implementing the intervention (rounding time and challenges)	
	Percentage reduction of total daily cost of PIMs		
Wouters H. [56]	Visit to outpatient clinics		
	Visit by elder care physician		
	Consultation by other health care professionals		
Zechmann S. [57]	Rate of hospitalization	Time consumption due to the intervention, by the practice nurse and by PCP	Frequency of discrepant decisions between GP and patient

Legend: primary outcomes are in bold.

Abbreviations: *BZD* Benzodiazepines, *CP* Clinical pharmacist, *GP* General practitioner, *PCP* Primary care physician, *PIMs* Potentially inappropriate medications

A total of three studies (8.3%) presented all four types of outcomes (medication, clinical, system, and implementation) [40, 55, 57], and 10 studies (27.8%) presented three types of outcomes: medication, clinical, and system outcomes for eight studies (22.2%) [28, 29, 32, 38, 42, 45, 46, 56], medication, clinical and implementation for one [49], and medication, system, and implementation for another [34]. These elements are described in the Table 2. Finally, 16 outcomes (*n* = 12; 33.3% of studies) were classified as "other outcomes" [22, 26, 28, 29, 34, 37, 41, 42, 44, 47, 49, 57].

The primary outcome was a medication outcome in almost three out of four studies (*n* = 26; 72.2%) [22–24, 27–36, 39, 43–45, 49–57]. In most studies, this outcome type focused on the number of drugs deprescribed or the number or percentage of patients for whom the deprescribing intervention was successful. The choice of medication outcomes was rarely justified or explained, and the methods for collecting these data were poorly developed. Indeed, the data were mostly collected from the patients’ charts, pharmacy dispensing databases, or GP reports but we had no further details. In some cases, this data was self-reported by the patients.

The primary outcome was clinical in five studies (13.9%), whether measuring cognitive function (*n* = 2; 5.6%) [41, 47], falls (*n* = 2; 5.6%) [25, 42], or delirium severity (*n* = 1; 2.8%) [26]. The majority of studies (*n* = 24; 66.7%) that included one or more clinical outcomes selected at least one outcome that was directly and specifically related to the drug deprescribed [24–26, 28, 29, 32, 35, 36, 38–42, 45–49, 52–57]. However, as with medication outcomes, the choice of the clinical outcomes was rarely justified. The other primary outcomes could be system outcomes (*n* = 1; 2.8%, cost saving of deprescribing) [40], both clinical and system outcomes (*n* = 1; 2.8%, unplanned hospital admission and death) [46], or other outcomes (*n* = 1; 2.8%, proportion of patients who initiated a discussion with a healthcare professional about deprescribing) [37]. The implementation outcomes were never primary outcomes. Two studies (5.6%) did not specify the primary outcome [38, 48].

## Discussion

The majority of studies included in this systematic review targeted potentially inappropriate medications in older adults. The follow-up periods of the studies were very heterogeneous and the interventions most often included a medication review carried out by a pharmacist or a multidisciplinary team. The majority of studies presented at least one medication outcome, which was very often the study's primary endpoint, making it possible to conclude whether or not the deprescribing intervention was successful. Many of the studies presented at least one clinical outcome, but the choice of outcome was very rarely justified or applied, as was the method of measurement. All studies included in this review were published before the USDeN recommendations were published. The comparison of deprescribing implementation trial outcomes with USDeN recommendations [20] shows that work will be needed in the coming years to harmonize practices and increase the level of evidence of deprescribing studies. Indeed, only 3 studies presented the 4 types of outcomes recommended [40, 55, 57]. Two of these 3 studies targeted the deprescribing of PIMS in older adults [55, 57], and all 3 interventions were medication reviews [40, 55, 57]. Therefore, it was not shown that drug-specific or multidisciplinary interventions adhered more to the guidelines than other interventions. The studies to date are not very comparable because of their characteristics and the heterogeneity of the selected outcomes. It would be interesting to repeat this work in 10 years' time to measure the impact of these recommendations.

### The need to consider outcomes selection in deprescribing trials

The USDeN recommendations state that clinical outcomes should be the primary outcome assessed in deprescribing trials. The conclusion made by Gnjidic and Reeve in 2020, pointing out that these studies are typically focused on the success of the intervention, and, therefore, the primary outcomes are often the proportion of participants who stopped a medication [11], supports our findings. Thus, it seems crucial to harmonize practices and terminology regarding the primary outcome of deprescribing trials to increase the levels of evidence in these studies.

As pointed out by the USDeN, it is essential that the outcomes that quantify drug switching benefit from more standardized definitions. For example, is it more relevant to measure the number of potentially inappropriate medicines prescribed to older adults or to measure the number of patients with at least one potentially inappropriate medicine? These results may vary depending on how they are presented. It is also critical to consider the substitution of a drug for another substance, whether or not it is a medication, when discussing the success of an intervention. This pragmatic aspect of the impact of deprescribing is all too often not measured in the included studies of this review. For example, when successfully deprescribing a BZD, can the intervention be considered a success if the patient has switched to other substances such as alcohol, cannabis, or doxylamine? The same applies to deprescribing an opioid and new or increased cannabis use. It should be noted that it is often difficult to measure these outcomes with any rigor, as they are often self-reported by the patient. In this regard, the data found in the patient’s medical records or the health insurance databases should always be considered with caution. Indeed, it is a common occurrence for drugs dispensed without a prescription to not be recorded, or a drug dispensed to not be a drug taken by the patient. Thus, cross-referencing several sources, such as official databases with patient-reported data, provides more robust indicators of the effectiveness of the intervention.

Deprescribing trials need to include other categories of outcomes (clinical, system, implementation), as specified by the USDeN. These recommendations do not address the number and types of outcomes to be selected for trials, nor do they address the need to measure medication, clinical, system, and implementation outcomes. In our opinion, this choice depends on the objectives of each study and the drugs targeted by the deprescribing intervention. The acceptability of the measurement of many patient-reported outcomes must also be considered to avoid patient fatigue during the assessment and thus biased results. However, it appears to us to be a priority to associate a clinical outcome to assess the clinical impact of the drug deprescribed and to ultimately justify the intervention. For example, Kersten et al*.* [41] chose to measure mouth dryness after deprescribing anticholinergic drugs in older adults. The choice of this outcome is questionable in terms of the risk/benefit of the drug and the patient's quality of life. The patient's quality of life or the measure of a functional dimension appear to be more relevant clinical outcomes. In this case, the timing of the measurement and the appropriateness of the instrument used should also be considered and justified.

In our review, only seven studies out of 36 proposed an evaluation of implementation outcomes [22, 34, 37, 40, 49, 55, 57], mostly assessing the time required for the intervention or the perception of healthcare professionals regarding deprescribing. Cost assessment could be classified as an implementation outcome or a system outcome. Implementation science was born out of the need to effectively translate research findings into practice in order to bridge the gap between research and practice [62]. This involves measuring different criteria, such as acceptability, adoption, feasibility, or even sustainability in real life [63]. Our results show that this area of research on deprescribing is still in its early stages and that there is an urgent need to integrate implementation criteria into deprescribing trials. This is what many researchers in this field [62, 64] have called for, leading to the publication of recommendations [65, 66], tools [67], or frameworks [68, 69] for which the objective is to translate deprescribing, or as some call it "de-implementation" [70], into practice. In addition to the other outcomes measured during clinical trials, these implementation outcomes guarantee that the interventions can indeed be implemented and not just remain in the literature.

### Going beyond the confines and extending the follow-up time

The majority of the included studies were conducted in nursing homes, hospitals, or rehabilitation hospitals. These settings can be an obstacle to deprescribing, particularly for the deprescribing of BZDs or antipsychotic drugs in an anxiety-inducing environment. Performing deprescribing upstream, *i.e*., in primary care, could prevent patients from being institutionalized or hospitalized. However, it should be kept in mind that having the patient under clinical supervision is a key element in the feasibility of a deprescribing trial. The hospital or tertiary care setting allows withdrawal events to be followed and monitored more readily. In addition, multidisciplinary teams in these settings are also a lever for deprescribing. Giving greater consideration to more frequent implementation of deprescribing trials in real life should be undertaken because hospitalization or institutionalization only affect a minority of patients, which leads to lower generalizability. In this review, the reported follow-up period was very heterogeneous, and the timing of measurement was rarely justified. This heterogeneity may be related to the time required to deprescribe certain classes of drugs, particularly for medications used on a long-term basis. For example, deprescribing a drug that does not cause withdrawal syndrome (e.g., aspirin in primary prevention) is certainly faster than deprescribing a BZD or an opioid. It would appear that in most studies these follow-up timelines were chosen based on the study feasibility, which appears to be a limitation to the success of the intervention. A long follow-up period (more than 12 months) allows for conclusion that the intervention is safe and sustainable. However, it makes it likely that patients will experience events leading to treatment resumption or to be lost to follow-up. Hence, it is essential to justify the choice of the follow-up period and the measure of the intervention according to the objective of the study. This is in line with USDeN recommendations stating that clinical outcomes are often measured too early or too late concerning the clinical effect. Finally, when it comes to deprescribing PIMs, it is necessary to homogenize practices and establish a consensus on the classification of inappropriate drugs in older adults, as different studies use different lists to select included patients.

### Strengths and limits

The main strengths of this study lie in the quality of the articles included. Randomized controlled trials represent the gold standard of trials, for which there is a great deal of methodological reflection. In addition, three investigators independently evaluated each title and abstract. We acknowledge several limitations of this review. First, we only included studies published in the past 10 years. This choice was made because the majority of deprescribing trials have been conducted over the past decade [71, 72]. Secondly, our search equation only queried the titles in the databases. We made this choice by constructing a search equation with numerous terms directly related to deprescribing. Indeed, researchers often devote a great deal of effort to choosing appropriate titles that reflect the main objective and design of their studies. Thirdly, we only included studies with usual care as a comparator, which appeared to be the most relevant to us for adaptation in primary care. Fourthly, we did not analyze the references of the selected articles, which could have resulted in the omission of some eligible articles. Finally, as with any review, a publication bias is likely due to the frequent non-publication of non-significant results [73].

## Conclusion

In conclusion, this analysis confirmed our hypotheses regarding the importance of harmonizing deprescribing study methods to generate usable clinical evidence. There is a need to propose recommendations for real-life deprescribing trials, starting with the integration of deprescribing as soon as prescriptions go beyond the appropriate use of the drug, with sufficiently long follow-up periods, relevant outcome measurement times, and implementation measures allowing reproducibility of interventions. Switching from a discontinued drug to another drug or non-drug substance needs to be more extensively measured to conclude the success of deprescribing interventions. Implementation outcomes need to be developed and specified to facilitate the application and the reproducibility of these practices on a larger scale. Finally, clinical outcomes need to be justified and prioritized.

## Supplementary Information

**Additional file 1: Appendix S1.**

**Additional file 2: Appendix S2.**

## Data Availability

The datasets used and analyzed during the current study are available from the corresponding author upon reasonable request.
