# Supplementary material for: Outcomes in deprescribing implementation trials and compliance with expert recommendations: a systematic review
Source: BMC Geriatr. 2023 Jul 12;23:428. doi: 10.1186/s12877-023-04155-y (PMC10337166; doi:10.1186/s12877-023-04155-y)
Supplement: Supplementary file 1 — Additional file 1: Appendix S1. [file 12877_2023_4155_MOESM1_ESM.docx]

**Supplementary File S1. MEDLINE search strategy**

Equation

((ceas*[Title]) OR (cessation[Title]) OR (decreas*[Title]) OR (deprescrib*[Title]) OR (de-prescrib*[Title]) OR (deprescrip*[Title]) OR (de-prescript*[Title]) OR (discontinu*[Title]) OR (eliminate*[Title]) OR (reduc*[Title]) OR (stop*[Title]) OR (taper*[Title]) OR (substitut*[Title]) OR (withdraw*[Title]) OR (optimiz*[Title]) OR (remov*[Title]) OR (interrupt*[Title]) OR (step-down*[Title]) OR (restriction[Title]) OR (deintensification[Title]) OR (diminish*[Title]) OR (drop*[Title])) NOT (Smok*[Title]) NOT (Cigar*[Title]))

Article type

- Clinical Trials
- Randomized Controlled Trial

Date

- January 1st, 2012 to January 1st, 2022
